# Reshaping neurosurgical training: a novel simulation-based concept for structured skill acquisition and curriculum integration

**DOI:** 10.1007/s10143-025-03666-z

**Published:** 2025-06-20

**Authors:** Belal Neyazi, Amir Amini, Vanessa M. Swiatek, Klaus-Peter Stein, Ali Rashidi, I. Erol Sandalcioglu

**Affiliations:** 1 Department of Neurosurgery, Otto-von-Guericke University, Magdeburg, Saxony-Anhalt Germany; 2 Department of Neurosurgery, Otto-von-Guericke University Hospital, Magdeburg, Saxony-Anhalt Germany

**Keywords:** Construct validity, Transferability surgical skills assessment, Simulation training, Neurosurgical education, 3D-Printing

## Abstract

As neurosurgical caseloads decline, alternative training methods are essential for practical, hands-on training and technical skill acquisition. Physical simulators have the potential to enhance surgical training, but current models lack realism and fail to replicate the full surgical workflow. This study aims to develop and evaluate a novel, affordable microneurosurgical simulator designed for structured, effective and transferable skill acquisition. Based on previously established methodologies, we developed a novel high-fidelity yet cost-effective simulator that replicates the key steps of the neurosurgical workflow, including patient positioning, craniotomy, microsurgical dissection, clipping of aneurysms, tumor resection, and closure techniques. The simulator’s fidelity was validated through intense rheological testing and tactile evaluations by experienced neurosurgeons. It was subsequently implemented in a two-day microneurosurgical simulation course involving 12 neurosurgical residents from leading German institutions. Participants completed pre- and post-course evaluations. Objective evaluations of technical proficiency and surgical learning curves were conducted using a newly developed tool—the Objective Structured Assessment of Neurosurgical Skills (OSANS). Participants rated the simulator highly for anatomical accuracy and tactile realism, with 95% considering it “highly realistic.” Objective assessments revealed significant improvements in technical skills, including craniotomy precision and dural closure. Confidence in performing complex neuro-oncological and vascular procedures increased by 40%. Incorporating simulation-based training into neurosurgical curricula can enhance resident education, improve skill acquisition, and promote patient safety. This presented cost-effective, reusable simulator bridges gaps in neurosurgical training by enabling realistic and repetitive practice.

## Introduction

The acquisition of technical skills in neurosurgery remains one of the most challenging aspects of surgical training due to the intricacy of neuroanatomy and the high stakes of neurosurgical procedures. Traditional neurosurgical education has long followed the Halsted Ian principle of “see one, do one, teach one” [1, 2] an approach that is increasingly regarded as inadequate in modern contexts where surgical caseloads are steadily decreasing [3, 4]. These challenges necessitate alternative training platforms that provide realistic, reproducible, and accessible opportunities for skill development. In recent years, simulation-based training has gained recognition as a valuable and widely accessible alternative. However, there remains a lack of standardized criteria for assessing the quality and integration of simulators into neurosurgical education and training [5].

Physical simulators offer considerable promise for advancing surgical training by enabling practical, hands-on learning experiences. However, current phantom simulators are markedly limited by high acquisition costs, insufficient tactile and anatomical realism, and restricted usability [6–8]. These shortcomings include the inability to replicate a complete surgical workflow, support individualized planning, or provide a safe environment for learning from mistakes. Consequently, such simulators fall short of delivering structured and effective training opportunities. To address these gaps, we developed a novel hands-on microsurgical simulator for cranial procedures, including the management of cerebral aneurysms and the resection of intra- and extra-axial brain lesions. Using freeware for segmentation and post-processing of pathology-specific medical imaging data, combined with additive manufacturing processes, the simulator can be used for deliberate focused training of basic microneurosurgical skills and approaches as well as the simulation of complex procedures such as the microsurgical management of cerebral aneurysms and tumors.

Leveraging this simulator, we conducted a multicentric simulation course involving neurosurgical residents from leading institutions across Germany, providing hands-on training in advanced neuro-oncological and neuro-vascular procedures. This presented study describes the development and validation of the simulator, the course structure, and its impact on participants’ performances. Significant emphasis was placed on both subjective and objective evaluations of the course and the simulator, with comprehensive assessments conducted to gauge the course’s impact on the surgical learning curve. By providing a detailed framework for the design, evaluation, and organization of the course, along with guidelines for simulator integration, we advocate for the broader adoption of simulation-based training and the development of standardized protocols in neurosurgical education.

## Materials and methods

### Data acquisition

This study was approved by the local ethics committee of the Medical Faculty of the Otto-von-Guericke University. Computed tomography angiography (CTA) and magnetic resonance imaging (MRI) datasets of a healthy 35-year-old male were used for the segmentation and reconstruction of the skull, brain, cerebral vessels and associated anatomical structures.

### Construction of the simulator

**Fig. 1:**
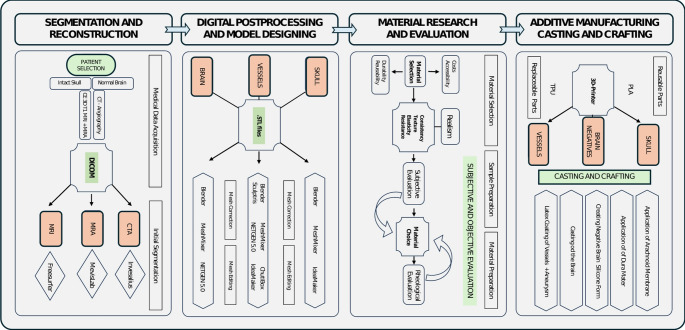
Structured workflow detailing the reconstruction, digital editing, additive manufacturing, material research, and crafting process of the simulator

The construction of the simulator was based on previously established methodologies [9, 10]but involved a more complex design compared to previous models as it incorporates the entire brain, cerebral vessels, meninges and various pathologies. This process was executed in four stages, summarized in Fig. 1.

#### Segmentation and digital reconstruction

The skull was segmented from the CTA dataset using *InVesalius3* (Centro de Technologic da Informação Renato Archer (CTI)) based on greyscale boundaries. The 3D mesh was post-processed in *MeshMixer 3.5* (Autodesk Inc., https://meshmixer.en.softonic.com/mac) and *Blender* (Blender Foundation, www.blender.org) to divide the skull into a reusable base and multiple replaceable regions aiming to minimize material costs and printing time.

The brain was segmented from the patient’s high-resolution contrast-enhanced T1 weighted MRI dataset using the open-source neuroimaging analysis and visualization toolkit *FreeSurfer* (Harvard University, https://surfer.nmr.mgh.harvard.edu).

The falco-tentorial complex and the circle of Willis (CoW) were segmented from contrast-enhanced T1 weighted MRI datasets using the medical image processing tool *MeVisLab 2.6.2* (MeVis Medical Solutions AG, Bremen, Germany, www.mevislab.de). Threshold-based techniques ensured accurate extraction of vascular structures.

#### Digital post-processing and designing

Skull: Each replaceable part of the skull was designed with integrated locking mechanisms using *Blender’s* Boolean functions and *MeshMixer’s* customization tools. This ensured a seamless connection between the parts and the base while maintaining anatomical accuracy.

Brain: While *FreeSurfer’s* automated segmentation accurately captured cortical surfaces, deeper anatomical features of the brain such as the Sylvian fissure and specific anatomical boundaries required manual refinement and post-processing performed in *Blender*, where the 3D mesh of the brain was meticulously separated along anatomical landmarks.

Cerebral Vessels: The 3D mesh of the CoW was hollowed out using *ChituBox* (CBD Technology Co., https://www.chitubox.com/en/index) and further refined in *Blender* and *Sculptris 1.02* (Pixologic, Inc., www.sculpteo.com) to ensure anatomical fidelity and compatibility with optional blood perfusion simulations. A similar approach was employed to reconstruct large veins and sinuses. Using *Blender’*s modeling and sculpting features, aneurysms of different sizes and angulations were added digitally to the 3D mesh of the CoW.

#### Substudy: simulating the living brain

- *Material selection and preparation*.

To identify the most suitable material for simulating the living brain tissue as encountered during surgery, which significantly differs from the tactile properties of cadaveric brain tissue [11, 12]we evaluated a variety of materials commonly used in biomedical simulations including gelatin 260 bloom, polymethyl acrylate (PMA), and polyvinyl alcohol (PVA) at various concentrations. Each material was selected based on its established use in medical simulations and its ability to mimic rheological properties similar to the living brain tissue. For gelatin, solutions were mixed at 10%, 15%, and 20% concentrations (weight/volume) to evaluate the impact of density on tactile realism. PMA and PVA samples were similarly prepared at concentrations of 5%, 10%, and 15%. All samples were allowed to cure at room temperature for 24 h to achieve stable mechanical properties and ensure consistency.

**Fig. 2:**
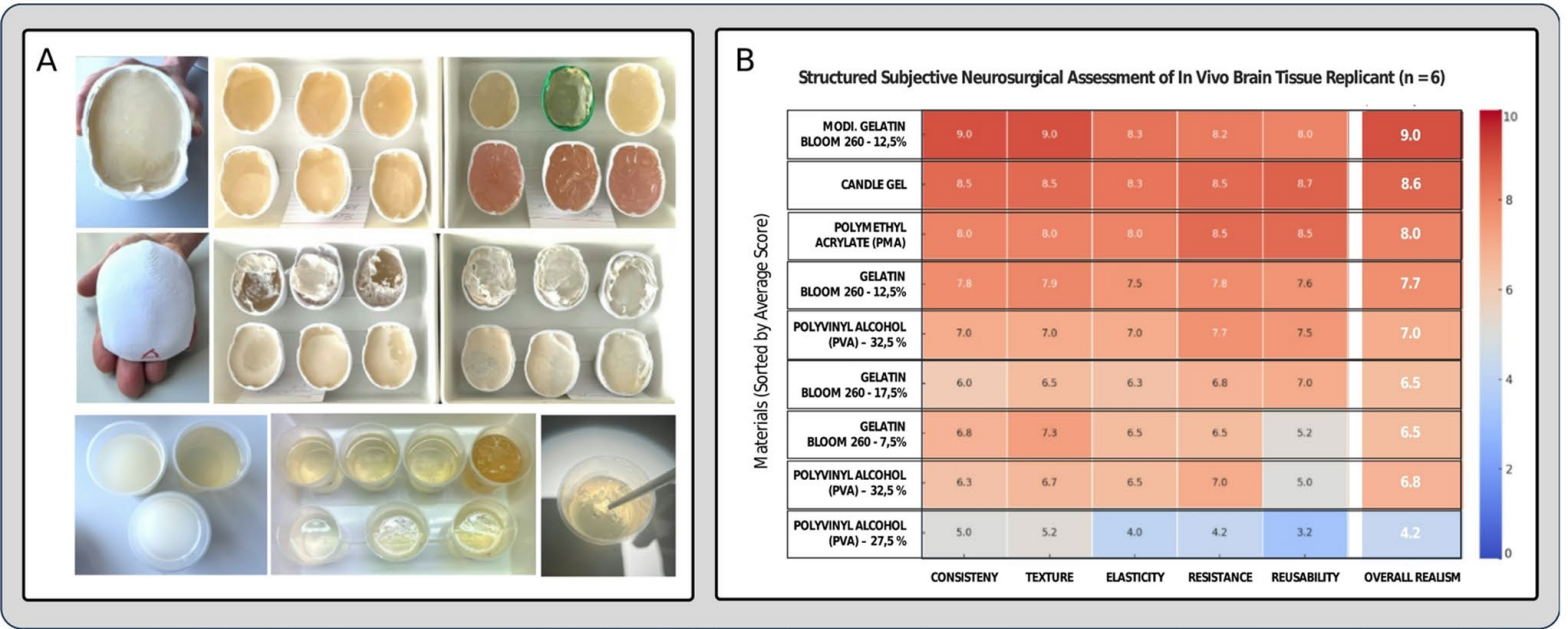
**(A)** Evaluation process including the dissection, resection and suction of materials presented in skull-shaped containers with surrounding meningeal layers. **(B)** Results of the subjective assessment of tested materials by experienced neurosurgeons (*n* = 6) regarding their tactile properties derived from a 10-point Likert scale

To enhance the realism of the evaluation process, the material samples were presented in skull-shaped containers printed using polylactic acid (PLA) filaments. These containers were designed to mimic the structural features of the cranial vault, including realistic bony surfaces (Fig. 2, A). Each container also included simulated meninges created from thin silicone membranes to replicate the anatomical layers typically encountered during neurosurgical procedures. This setup allowed for a more realistic representation of tactile interactions between the neurosurgeon, the “brain tissue,” and the surrounding structures.

- *Material evaluation*.

The evaluation process involved six experienced neurosurgeons, each with a minimum of 10 years of surgical experience in neuro-oncology and vascular neurosurgery. The neurosurgeons were blinded to the identity and composition of the material samples to ensure unbiased assessments. Each neurosurgeon tested the anonymized specimens in a controlled laboratory setting using microsurgical instruments.

The materials were evaluated under consistent laboratory conditions to eliminate variability in lighting, temperature, and other environmental factors. The skull-shaped containers were mounted on a fixed support to simulate the stability provided by a surgical head clamp.

The neurosurgeons rated each material on a 10-point Likert scale for realism in tactile feedback, response to dissection, response to suction, and interaction with surrounding structures. (Fig. 2, B) These properties were evaluated to determine how closely the materials mimicked the rheological and mechanical behavior of living brain tissue and pathological processes encountered during surgery.

- *Results of the substudy*.

Based on quantitative ratings and qualitative feedback, the modified gelatin 260 bloom at 12.5% concentration achieved the highest mean score of 9.2 ± 0.3, significantly outperforming other materials in all evaluated categories.

- Tactile feedback: 9.5 (± 0.2).
- Response to dissection: 9.3 (± 0.4).
- Response to suction: 8.9 (± 0.5).
- Interaction with surrounding structures: 9.2 (± 0.3).

This material offers the unique advantage of combining anatomical realism, rheological accuracy, and long-term stability, overcoming the limitations of previous gelatin formulations. Although price and accessibility were not factors in the neurosurgeons’ evaluations, as they were blinded to the material identities, the modified gelatin 260 bloom also emerged as the most cost-effective option among the tested materials.

#### Stage IV: additive manufacturing, crafting and casting

**Fig. 3:**
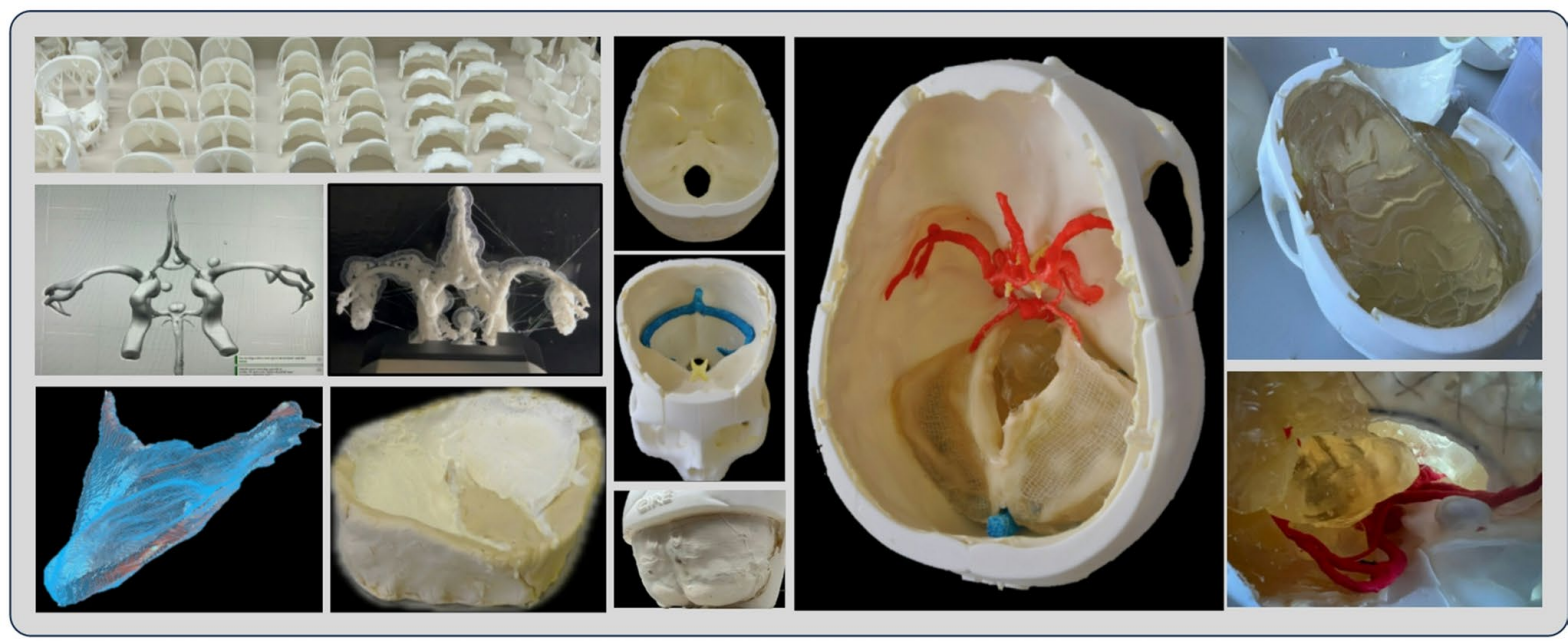
Results of the reconstruction, digital postprocessing and 3D printing of the skull, brain and vessels using PLA and elastic TPU filaments. The assembly process of the simulator begins with the application of the dura mater followed by the placements of the CoW, venous sinuses, the cerebellum, brainstem, tentorium cerebelli, and the temporal and fronto-parietal lobes, each containing various lesion

All components the brain and the skull were printed using a Raise3D Pro2 dual-extrusion 3D printer (*Raise 3D Technologies*,* Inc*.) with 1.75 millimeters (mm) white PLA filaments (*PrimaCreator*) at temperatures between 195 and 235 °C (degree Celsius) with a layer height of 0.25 mm. The heatbed temperature was set between 55 and 75 °C. For the skull parts, an infill density of 15% was selected to ensure stability and simulate the rigidity differences between compact and cancellous bone during the drilling process. The brain was printed in parts to facilitate the negative molding and casting process later. All vascular models, including the CoW and aneurysms were printed using elastic thermoplastic polyurethane (TPU) filaments (*NinjaTec*) with a nozzle temperature of 235 °C and a heatbed temperature of 40 °C. The printing speed was reduced to 20 mm/s to maintain accuracy and prevent warping (Fig. 3). Negative molds of the brain components were created using silicone (*Wagnersil)* with a hardness of 22 Shore A. The modified gelatin 260 bloom (12.5% concentration) was melted at 95 °C and poured into the silicone molds.

Intracerebral pathologies such as meningiomas, cavernomas and glioma were crafted manually using marzipan or licorice and placed at desired locations within the mold during the casting process using removable needles for stabilization. After cooling for 30 min, the brain components were demolded and assembled to form the complete brain structure.

The elastic models of cerebral arteries and veins were coated with two thin layers of colored liquid latex to enhance visual and tactile realism. The arachnoid mater was simulated by applying a mixture of crystal-clear synthetic resin adhesive and diluted latex onto the glue web surrounding the brain components. Liquid latex (*Lilatex*) was applied to the inner surfaces of the skull to replicate the dura mater, adhering to the skull base with a coalescing agent. We used simple clay mold to cast the tentorium cerebelli using a combination of elastic 3D mesh fabric and liquid latex. The resulting models were usable after a drying process of 12 h at room temperatures.

### Study design

#### Participants

A total of 12 participants, consisting of second- and third-year neurosurgical residents, were recruited from six neurosurgical departments of different university hospitals in Germany: Dresden, Mannheim, Technical University of Munich, Essen, Berlin, and Magdeburg. The cohort comprised an equal gender distribution, with six male and six female residents between the ages of 27–32. To ensure diversity and reduce institutional bias, the participants were randomized into six groups of two. Participants were required to adhere to standard neurosurgical protocols and were encouraged to interact with their group partners for intraoperative decision-making.

#### Simulation setup

**Fig. 4:**
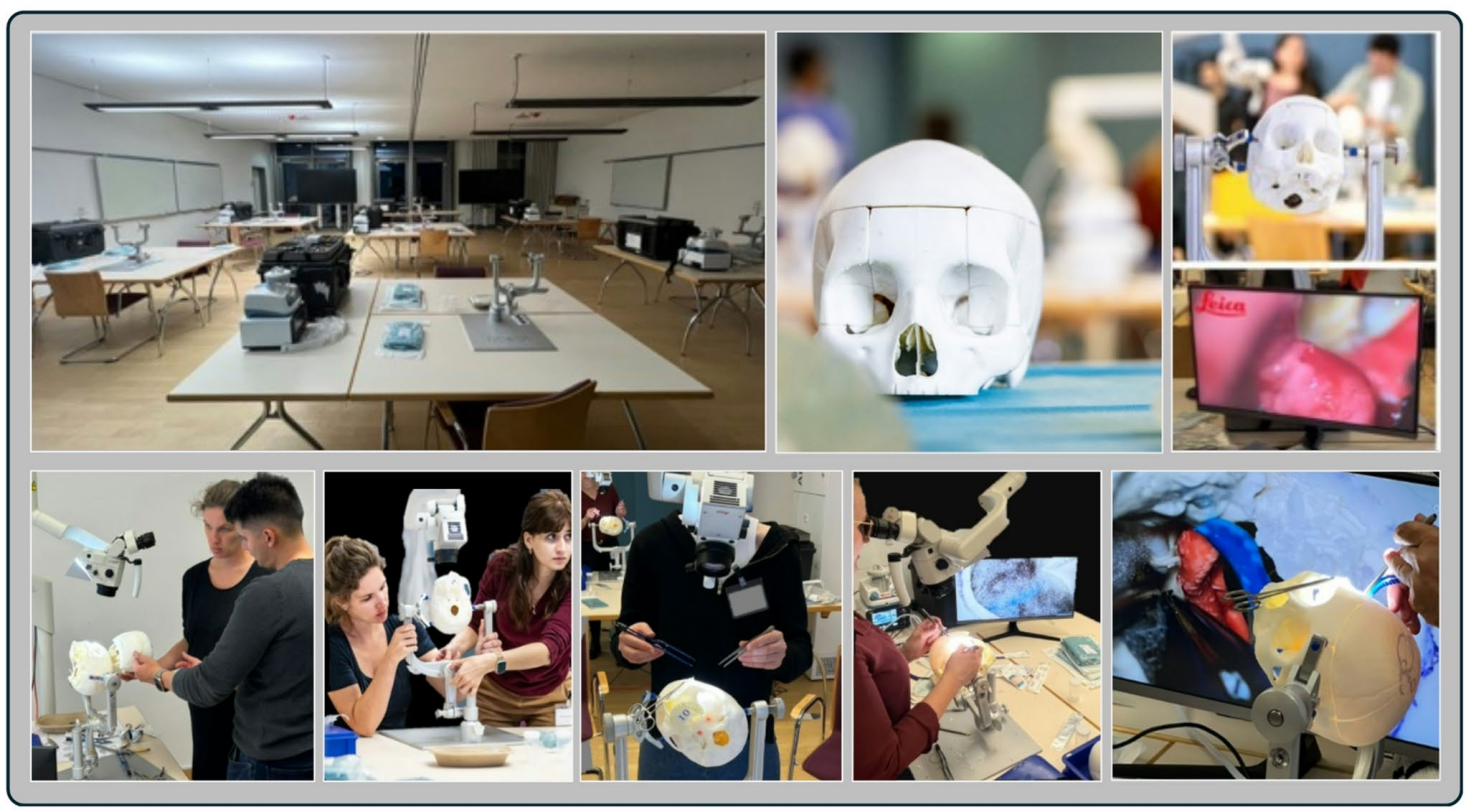
Simulation setup with a total of six simulation stations equipped with neurosurgical microscopes, microsurgical instruments, suction- and three-point fixation devices

Six neurosurgical simulation stations were provided for the study (Fig. 4). Each station was equipped with a microneurosurgical microscope by *Leica* (Leica M320 Surgical Training Microscope, www.leica-microsystems.com), a monitor displaying the surgeon’s view, a three-point skull fixation device by *DORO* (DORO QR3 Skull Clamp System, www.blackforestmedical.com) and a full craniotomy set (NSK www.nsk-surgery.com) including a *Primado2* high-speed drilling system and a surgical suction device (MEDUTEK KATASPIR 20 eco, www.medutek.com) as well as a full set of microneurosurgical instruments, including aneurysm clips (Aesculap Yasargil, ww.aesculapusa.com). Each station was equipped with two phantom simulators accompanied by detailed information sheets specifying the anatomical sites, sizes or angulations of the targeted tumors and aneurysms.

#### Simulation process

The simulation course spanned two days:

**Day 1**: The first part of day 1 focused on foundational skills. Following a comprehensive introduction on neurosurgical approaches and techniques, the participants started the simulations with head positioning, fixation, burr hole and craniotomy placements. The second part involved the microsurgical neurovascular session, where participants practiced the clipping of an aneurysm of the middle cerebral artery bifurcation via a standard pterional approach.

**Day 2**: Concentrated on advanced techniques in skull base surgery and tumor resection. Participants performed debulking and excision of a tumor located in the cerebellum via a retrosigmoid approach while ensuring the preservation of critical adjacent structures.

Performance metrics, which focused on technical proficiency, decision-making, and procedural accuracy, were complemented by comprehensive debriefing sessions. During these sessions, participants reviewed their performance with faculty members using video recordings of their procedures, receiving targeted feedback that highlighted strengths and areas for improvement.

#### Assessment and feedback

Each participant took part in an online survey designed to assess their perceptions of the simulator’s usability, educational usefulness, and perceived accuracy. Participants rated their agreement with these statements on a 5-point Likert scale, providing subjective feedback on the simulator’s effectiveness and realism.

To evaluate the participants’ surgical proficiency and learning curves, we adapted the Objective Structured Assessment of Technical Skills (OSATS) [13] tool to better suit the specific demands of neurosurgical procedures, particularly aneurysm clipping and tumor resection. The resulting instrument, named the “Objective Structured Assessment of Neurosurgical Skills” (OSANS), is a standardized evaluation tool consisting of eight criteria. (Table 1) These encompass key dimensions of neurosurgical performance, including surgeon posture, precision in instrument and tissue handling, and the quality of target exposure. Experienced neurosurgeons from our department employed this tool to assess each participant’s performance based on the following criteria:

- **Live performance analysis**: skills as assessed during the course.
- **Video analysis**: recordings from the surgical microscopes.
- **Phantom simulator analysis**: inspections of disassembled simulators post-course.

**Table 1:**
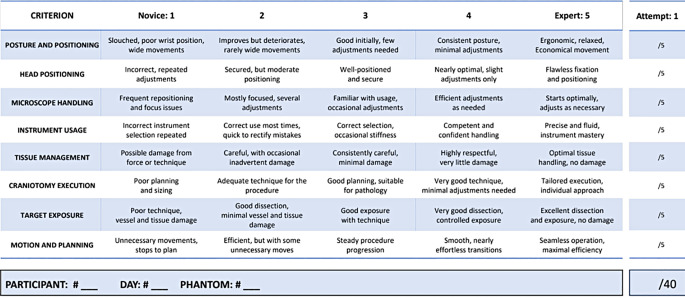
The Objective Structured Assessment of Neurosurgical Skills (OSANS)

## Results

The construction of the phantom simulators incorporated a four-stage process that ensured anatomical accuracy and functionality for simulating neurosurgical procedures.

### Subjective evaluation

**Fig. 5:**
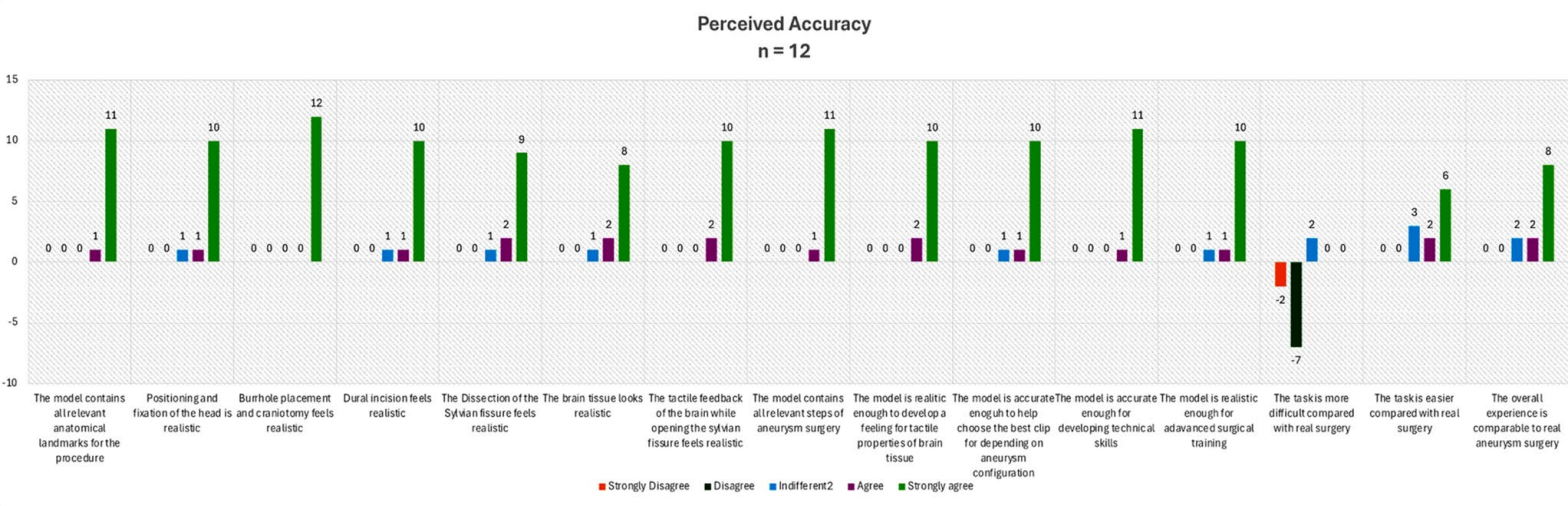
Results of the perceived accuracy of the simulator derived from a 5-point Likert scale

The perceived accuracy of the neurosurgical simulator was highly rated by the participants, with most expressing strong agreement across key aspects. The simulator’s realism, including anatomical landmarks, burr hole placement, and craniotomy, received unanimous positive feedback, highlighting its effectiveness in replicating surgical procedures. Features such as brain tissue appearance, tactile feedback, and dissection of the Sylvian fissure were also rated favorably, emphasizing the simulator’s suitability for hands-on training. While the simulator’s realism in advanced surgical steps was appreciated, opinions varied regarding task difficulty compared to real surgery. Some participants found the tasks more challenging, while others deemed them easier, reflecting a subjective perception of difficulty (Fig. 5). 92% of participants found the simulator intuitive and effective for procedural training, and over 95% agreed that the simulators improved their understanding of anatomical relationships and surgical techniques.

**Fig. 6:**
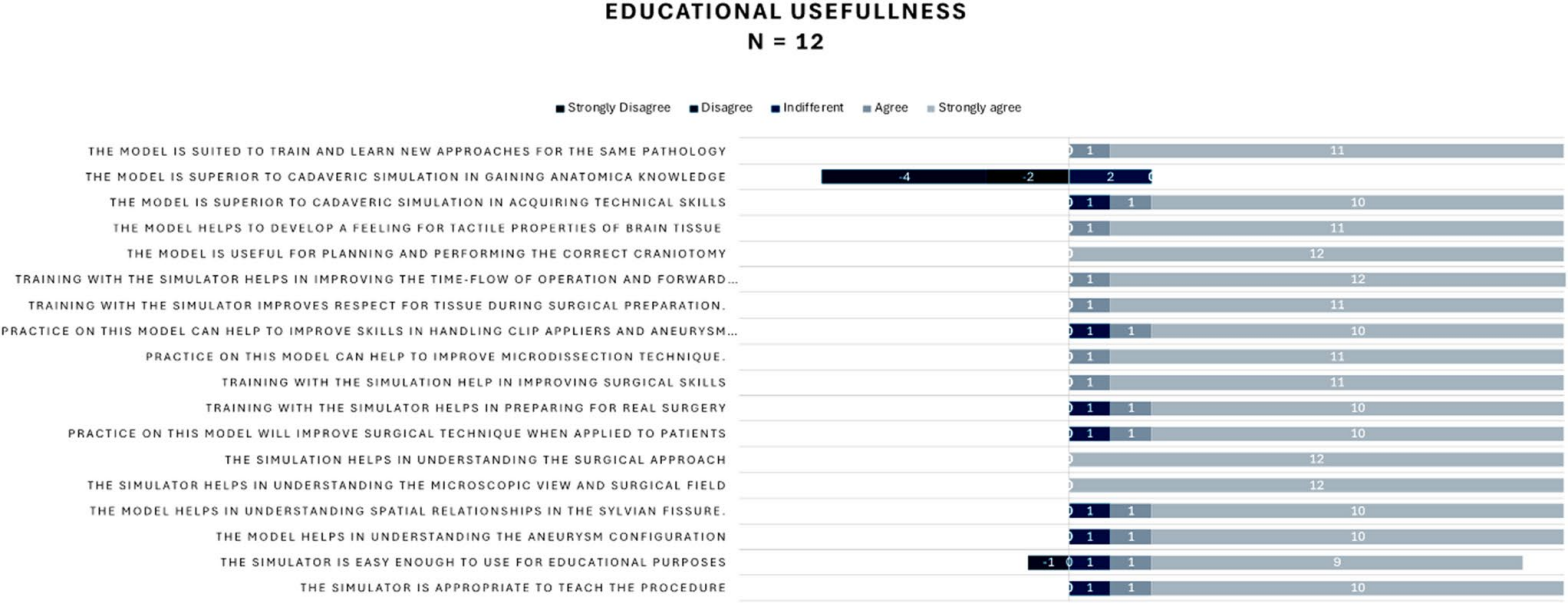
Results of the subjective evaluation of the simulator’s educational usefulness by participants derived from a 5-point Likert scale

Participants overwhelmingly agreed that the simulator is appropriate for teaching procedures, easy to use for educational purposes, and aids in understanding anatomical configurations and spatial relationships, such as in the Sylvian fissure. (Fig. 6) Participants also emphasized its effectiveness in replicating microscopic views, surgical fields, and approaches. The simulator was found to enhance surgical preparation, improve microdissection techniques, and develop skills in handling clip appliers and performing aneurysm clipping. Moreover, it was noted for improving respect for tissue and aiding in operational planning and execution. A notable point was the recognition of the simulator as superior to cadaveric models in acquiring technical skills and anatomical knowledge. Participants reported increased confidence and preparedness for real surgical scenarios, confirming the simulator’s value in bridging the gap between theoretical knowledge and practical application. Overall, the simulator was highly rated as a tool for training and learning new approaches, demonstrating significant educational value in surgical training.

### Objective performance assessment

**Fig. 7:**
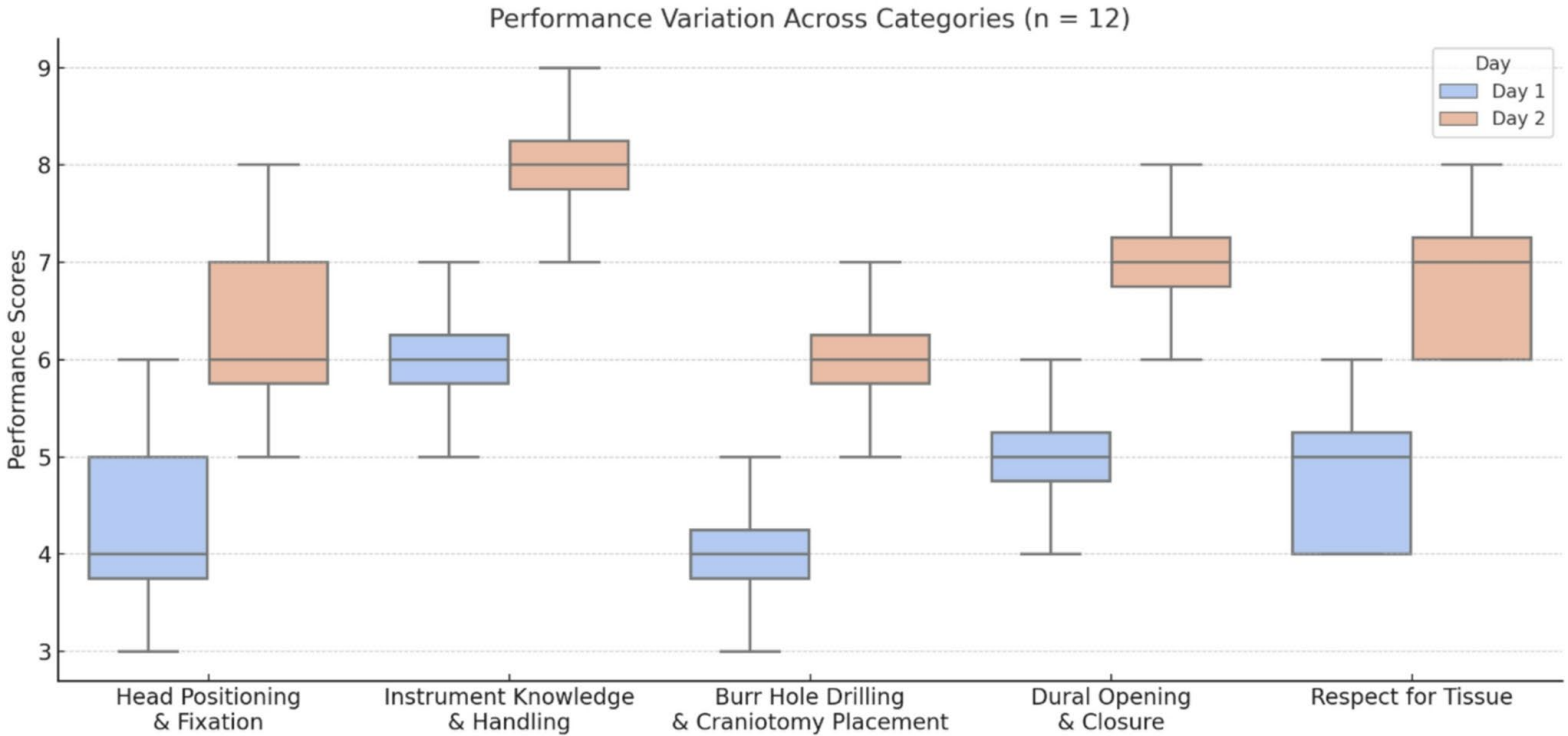
Results of the objective assessment of technical skills from day and day 2 in fundamental areas showing a significant improvement among all participants and across all categories. Based on the simulator’s high construct validity, a high predictive validity can be observed

The simulator demonstrated a high construct validity by accurately reflecting users’ technical skill levels. On average, participants demonstrated a rapid and significant improvement in technical proficiency from day 1 to day 2 including head positioning, instrument handling, and tissue respect, attributed to enhanced familiarity with simulation techniques and iterative practice (Fig. 7). Overall, technical proficiency scores improved by 25% on average between day 1 and day 2. Time management and decision-making during tasks such as aneurysm clipping and tumor resection was markedly better on day 2, with refined strategies and time efficiency. Faculty evaluations confirmed participants applied feedback effectively, bridging knowledge gaps observed on day 1. The post-course analysis revealed an 85% success rate in completing complex tasks. Overall, the combination of objective observations and subjective participant feedback strongly suggests that the skills acquired and refined during this course are transferable to the operative setting.

### Cost analysis

The cost analysis for the simulator distinguishes between the material expenses for initial fabrication and the recurring cost per simulation. The purchase of a 3D printer is not included in this calculation, as prices vary depending on the model; however, any standard desktop 3D printer is sufficient for production. The simulator was designed with reusable and replaceable components to minimize both material consumption and printing time. Since each set supports multiple simulations and only the skull and brain components used for surgical access and simulation need periodic replacement, the cost per simulation amounts to approximately $3 USD (Table 2).

**Table 2:**
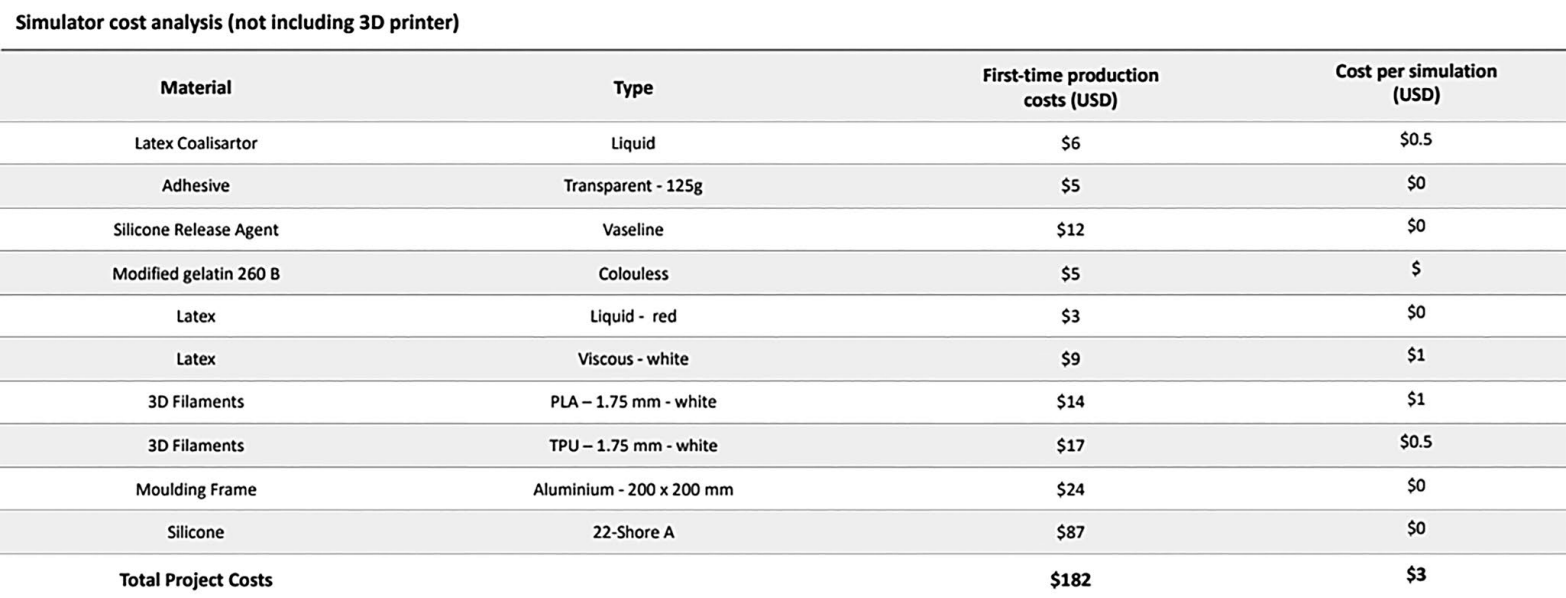
First-time production cost and continued operating expenses of the simulator

## Discussion

### Summary

High-fidelity simulation has emerged as an indispensable tool in surgical education, offering opportunities for deliberate practice in a risk-free environment. This study highlights the development and implementation of a novel high-fidelity simulator for neurosurgical training, focusing on its impact during a two-day course attended by neurosurgical residents. The simulator bridges critical gaps in existing training methods by providing an anatomically comprehensive, reusable, and cost-effective simulator capable of replicating nearly the entire neurosurgical workflow. Its unique design allows trainees to perform a variety of procedures, such as craniotomies, dural closure, and the handling of cerebral lesions and aneurysms, enhancing the transferability of acquired skills to real-world clinical settings.

### Interpretation

Neurosurgery is a field characterized by its technical complexity and demand for precision. Traditional methods, such as supervised practice in the operating room, increasingly fail to provide the exposure and experience necessary to acquire and hone technical skills for complex procedures. Furthermore, ethical considerations, patient safety concerns, and time constraints often reduce opportunities for residents to engage in high-stakes procedures. Cadaveric models, while providing valuable anatomical insights, lack the dynamic properties of living tissues, cannot simulate pathology-specific conditions, and are costly to acquire and maintain.

Recent years have witnessed a surge of innovation in simulation-based neurosurgical education, particularly with the integration of virtual reality (VR), augmented reality (AR), and 3D modeling techniques [14–16]. Neurosurgical hotspots such as the Barrow Neurological Institute, Mayo Clinic, Yeditepe University, and the University of Miami have produced significant contributions in this domain. Gurses et al. [17–19] introduced extended reality simulations and remote VR laboratories, enhancing accessibility to anatomical education during the COVID-19 pandemic and beyond, highlighting the value of immersive visualization tools for both trainee engagement and spatial understanding. Similarly, photogrammetry-based models [20–22] have enabled detailed 3D reconstructions of cadaveric specimens, providing high-resolution anatomical fidelity.

In a recent study by Hanalioglu et al. [23]patient-specific digital 3D models were shown to significantly improve the accuracy of neurosurgical planning among residents, particularly in tasks such as tumor projection and incision placement. Their work highlights the value of digital simulations not only as educational tools but also as objective assessment instruments for surgical planning proficiency.

However, these technologies often lack realistic tactile feedback, and the usage of microsurgical instruments, both of which critical for the development of technical skill. Currently available Phantom simulators may offer an alternative but are frequently constrained by incomplete anatomical representation, high production costs, and limited reusability. Many fail to simulate crucial aspects of neurosurgery, such as patient positioning, head fixation, and closure techniques, thereby limiting their effectiveness for comprehensive surgical training.

The presented simulator addresses these shortcomings in several ways. First, it provides a nearly complete cerebral simulation, including the entire brain, brainstem, cerebellum, cerebral vessels, and meninges, enabling the execution of a wide range of procedures. The simulator replicates the entire surgical workflow, from patient positioning and head fixation to craniotomy, tumor resection, and closure techniques. This holistic approach ensures that trainees are exposed to all critical steps of a neurosurgical procedure, better preparing them for real operative settings.

Second, the simulator’s design emphasizes cost-effectiveness and accessibility. By utilizing freeware for segmentation and digital post-processing, alongside readily available materials for 3D printing, production costs are significantly reduced. The modularity of the simulator, with reusable and replaceable components, further minimizes expenses and production time. This makes the simulator scalable and feasible for integration into residency programs, particularly in resource-constrained settings.

The educational value of the simulator was evident during the two-day course. Participants reported significant improvements in their confidence and technical proficiency, even within this short timeframe. Objective assessments demonstrated measurable enhancements in skills such as craniotomy placement, dural opening, and instrument handling. These rapid improvements underscore the potential of deliberate, repetitive practice using realistic simulation models.

A key strength of the simulator is its anatomical and tactile realism, as validated by experienced neurosurgeons. The materials used in its construction closely mimic the rheological properties of living tissues, providing realistic feedback during dissection, retraction, and closure. This level of fidelity is critical for ensuring that skills acquired during simulation training are transferable to the operating room.

Interestingly, the presented findings align with prior studies confirming the realistic tactile properties of gelatin 260 bloom at 12.5% concentration. However, earlier applications of this material were limited by its tendency to lose tactile properties after a few days, develop an unpleasant odor, and degrade rapidly, which rendered it unsuitable for long-term use. The modified formulation used in this study preserves the desirable tactile and rheological properties of the original gelatin while eliminating these limitations.

This innovation resolves a critical drawback, enabling the material to maintain its performance over an extended period without degradation. The combination of realistic properties and improved longevity positioned the modified gelatin 260 bloom as the optimal choice for simulating living brain tissue.

### Limitations and future directions

One notable limitation of this study is the relatively small number of participants (*n* = 12), which may affect the generalizability of the findings. While the cohort was purposefully selected from six different academic neurosurgical centers to ensure diversity and minimize institutional bias, the limited sample size restricts the statistical power of the study and may not capture the full variability in training needs or learning curves across broader populations of residents. Consequently, the observed improvements in technical performance and self-reported confidence, though encouraging, should be interpreted with caution. Future studies involving larger, multicentric cohorts will be essential to validate these preliminary findings, establish normative performance data, and further assess the simulator’s utility across different stages of training and institutional settings. Additionally, longitudinal studies will be necessary to evaluate the durability of skill acquisition and its transferability to the operating room over time.

The simulator, despite its many advantages, has limitations. It does not currently replicate dynamic intraoperative complications, such as aneurysm rupture or severe bleeding, which are critical components of vascular neurosurgery. Incorporating such scenarios in future iterations could further enhance its training value. Additionally, while the simulator closely mimics tissue properties, it cannot fully replicate the variability and complexity of live surgical environments, such as tissue response to retraction or dissection.

Future developments should focus on addressing these limitations. Dynamic feedback systems, possibly integrated with VR or AR technologies, could simulate intraoperative complications, offering trainees a more comprehensive learning experience. Furthermore, expanding the anatomical library to include rare or complex pathologies could increase the simulator’s versatility. Collaboration with industry partners or academic institutions could facilitate these advancements while maintaining the simulator’s cost-effective design.

## Conclusion

This study highlights the development and implementation of a novel high-fidelity neurosurgical simulator that bridges critical gaps in traditional and alternative training methods. Its affordability, anatomical comprehensiveness, and emphasis on replicating the entire surgical workflow make it a valuable tool for enhancing neurosurgical education. The rapid skill acquisition observed during the course underscores its potential to revolutionize training paradigms by enabling deliberate practice in a realistic and controlled environment. By addressing the limitations of existing methods and incorporating future improvements, this simulator can serve as a cornerstone for ensuring technical proficiency and advancing patient safety in neurosurgical training worldwide.

## Data Availability

No datasets were generated or analysed during the current study.

## Declarations

### Previous presentations

There are no previous publications to disclose for this manuscript.
